# Ontology Alignment Repair through Modularization and Confidence-Based Heuristics

**DOI:** 10.1371/journal.pone.0144807

**Published:** 2015-12-28

**Authors:** Emanuel Santos, Daniel Faria, Catia Pesquita, Francisco M. Couto

**Affiliations:** 1 Departamento de Informática, Faculdade de Ciências, Universidade de Lisboa, Portugal; 2 LASIGE, Faculdade de Ciências, Universidade de Lisboa, Portugal; Semmelweis University, HUNGARY

## Abstract

Ontology Matching aims at identifying a set of semantic correspondences, called an alignment, between related ontologies. In recent years, there has been a growing interest in efficient and effective matching methods for large ontologies. However, alignments produced for large ontologies are often logically incoherent. It was only recently that the use of repair techniques to improve the coherence of ontology alignments began to be explored. This paper presents a novel modularization technique for ontology alignment repair which extracts fragments of the input ontologies that only contain the necessary classes and relations to resolve all detectable incoherences. The paper presents also an alignment repair algorithm that uses a global repair strategy to minimize both the degree of incoherence and the number of mappings removed from the alignment, while overcoming the scalability problem by employing the proposed modularization technique. Our evaluation shows that our modularization technique produces significantly small fragments of the ontologies and that our repair algorithm produces more complete alignments than other current alignment repair systems, while obtaining an equivalent degree of incoherence. Additionally, we also present a variant of our repair algorithm that makes use of the confidence values of the mappings to improve alignment repair. Our repair algorithm was implemented as part of AgreementMakerLight, a free and open-source ontology matching system.

## Introduction

As ontologies become more prevalent and extensively used in domains such as biomedicine and geography, there is a growing need to automatically discover mappings (*i*.*e*., semantic correspondences) between them. This can be achieved through ontology matching in order to pursue the goal of the Semantic Web [1–5].

The widely used Web Ontology Language (OWL) provides a way to represent ontologies with well-defined semantics, and enables the definition of a set of mappings between ontologies, called an alignment, in the form of axioms. However, the union of the input ontologies *O*_*1*_ and *O*_*2*_ and the alignment *M* between them may cause unsatisfiable classes, *i*.*e*., classes that violate restrictions defined in the ontologies, such as disjointness. These incoherences can be easily detected using (automatic) logical reasoning, and must be addressed if the goal of the matching process is to obtain an integrated and logically sound knowledge source [6]. Solving them implies removing either restrictions from the ontologies or mappings from the alignment between them, with the latter being typically the preferred approach. The process of selectively removing (or altering) mappings from an ontology alignment with the goal of ensuring (or at least improving) coherence is called alignment repair [7].

Repairing an alignment is a complex process which requires: (1) identifying the unsatisfiable classes and/or properties; (2) identifying the set of mappings that cause unsatisfiabilities; and (3) removing (or altering) one or more of those mappings under a predetermined set of criteria.

The main paradigm of alignment repair, to which all repair algorithms adhere is the minimal impact paradigm. This consists on removing or modifying the smallest number of mappings possible from the input alignment so as to minimize the impact of the repair procedure [8]. The underlying assumption is that the alignment to be repaired is mostly correct, which is reasonable for both manually constructed alignments and automatically derived alignments using state-of-the-art ontology matching systems. Thus, by removing the smallest number of mappings possible, a repair algorithm is considered to be more effective because it will likely produce a more complete alignment. Additionally, under this paradigm, repair algorithms will tend to remove mappings that cause multiple incoherences, which are more plausible to be incorrect. Therefore, adopting this paradigm is also expected to yield more correct alignments.

It should be noted that while greater correctness and completeness are expected under the minimal impact paradigm, they are not ensured. There may very well be circumstances in which multiple incorrect mappings are in conflict with a single correct one, and that by removing the correct one a worst alignment is obtained. However, the problem is that it is impossible to ascertain the correctness of ontology mappings automatically, and if we knew it *a priori*, we wouldn't need to perform repair in the first place. Thus, we need to adopt heuristic criteria that enable us to select between conflicting mappings when performing repair, and the minimal impact paradigm is the soundest approach in a context of high quality ontology mappings.

In cases where the quality of the mappings is variable, it could make sense to adopt a variant of the minimum impact paradigm that minimizes the sum of confidence values of the removed mappings rather than the number of removed mappings.

To the best of our knowledge, there are currently only two other systems that perform alignment repair: LogMap [9,10] and ALCOMO [7]. LogMap is an ontology matching system that implements an efficient repair algorithm using incomplete reasoning-based techniques which typically produces coherent alignments. However, since it applies a local repair selection approach it often achieves coherence by removing or altering a suboptimal number of mappings. ALCOMO is an ontology alignment repair system that implements a complete repair algorithm, as well as a more efficient incomplete algorithm that typically produces alignments with a satisfactory degree of incoherence [11]. Because it applies a global repair selection approach, it cannot handle large ontologies efficiently.

In this paper, we propose a novel modularization algorithm that extracts the minimal fragments of the input ontologies necessary for detecting incoherences. We developed a global repair algorithm that aims to minimize the degree of incoherence under the minimal impact paradigm, and mitigates the scalability problem inherent to a global repair selection strategy using our proposed modularization algorithm. Our evaluation shows that our modularization algorithm produces significantly small fragments and that our repair algorithm has a smaller impact on the alignments than LogMap or ALCOMO, while obtaining an equivalent degree of incoherence. Finally, we present a variant to our approach that employs a filtering algorithm that uses the confidence values of the mappings to improve the quality of the repaired alignments. An earlier and draft version of this paper can be found in [12].

## Alignment Repair

A set of mappings that leads to unsatisfiable classes is referred to as *incoherent*. In the following formal definitions, ⊆ denotes the subsumption relation, ⊨ denotes the semantic consequence and *sigc*(*O*) denotes the set of classes in the signature of *O*.

Definition 1: A set of mappings M is incoherent with respect to *O*_*1*_ and *O*_*2*_ if there exists a class *x* in *sigc*(*O*_*1*_∪*O*_*2*_) such that *O*_*1*_∪*O*_*2*_ ⊭ *x* ⊆ ⊥ and *O*_*1*_∪*O*_*2*_∪*M* ⊨ *x* ⊆ ⊥.

The main approach to restore the coherence of a set is to selectively remove mappings from the set. The resulting set of mappings is called alignment repair.

Definition 2: Let *M* be an incoherent set of mappings w.r.t. *O*_*1*_∪*O*_*2*_. A set of mappings *R* ⊆ *M* is an alignment repair for *M* w.r.t *O*_*1*_ and *O*_*2*_ if *M* \ *R* is coherent w.r.t. *O*_*1*_ and *O*_*2*_.

There are two main approaches to repair: global repair, where the minimal impact is determined globally (*i*.*e*., by considering all conflicting sets of mappings before a repair selection is applied); and local repair, where the minimal impact is determined locally (*i*.*e*., for a subset of the conflicting sets of mappings). Since they ensure an overall minimum, global repair strategies generally produce better results than local repair strategies under the paradigm of minimal impact. However, they are computationally more demanding, and generally cannot handle very large ontologies.

Alignment repair can be computed using the state-of-the-art justification-extraction based approaches for debugging and repairing inconsistencies in OWL ontologies [13,14]. However, these techniques do not scale well with the number of unsatisfiabilities and/or classes.

One strategy to address the scalability issue is to compute an approximate repair using incomplete reasoning techniques (e.g. [7,10,15]). An approximate repair does not guarantee that the resulting alignment is coherent, but typically reduces the number of unsatisfiabilities. This strategy has been successfully applied to audit the UMLS metathesaurus [10].

Another strategy previously proposed to mitigate the scalability problem of alignment repair is ontology modularization, *i*.*e*., the extraction of a set of fragments from the ontologies that only includes the relevant information for performing alignment repair [9,14,16,17,18]. The goal is thus to ignore classes and/or properties from the ontologies that are unnecessary or redundant for the purpose of assessing coherence.

### LogMap

LogMap [9,10] is an ontology matching system that implements a local repair algorithm using reasoning-based techniques to achieve coherent alignments [9,10]. One particularity of its repair algorithm is that it splits equivalence mappings into two corresponding subsumption mappings. Thus, in addition to mapping removal, it supports the replacement of equivalence mappings by subsumption mappings. This strategy effectively decreases the number of mappings removed from the alignment, which is an issue for local repair strategies. However, based on the experimental evaluation, done by [6], of the OAEI Large Biomedical Ontologies track (which is also used in our evaluation), this strategy may alter the semantics of the mappings and lead to erroneous results. In order to handle large ontologies efficiently, LogMap implements the locality-based modularization proposed by Doran et al. [18]. LogMap encodes the input ontologies *O*_*1*_ and *O*_*2*_ as Horn propositional theories and exploits this encoding to subsequently detect unsatisfiable classes in an efficient and sound way during the repair process. These theories *P*_*1*_ (resp. *P*_*2*_) consists of the following Horn rules [19,20]:

- A rule *x*→*y* for all distinct classes *x*, *y* such that *x* is subsumed by *y* in *O*_*1*_ (resp. *O*_*2*_); subsumption relations can be determined using either an OWL 2 reasoner, or syntactically (in an incomplete way).
- A rule (*x*_*1*_∧…∧*x*_*n*_) →*y* for each subclass or equivalence axioms having the intersection of *x*_*1*_, …, *x*_*n*_ as subclass expression and *y* as superclass.
- Rules (*x*_*i*_∧*x*_*j*_)→*false* (1 ≤ i < j ≤ n) for each disjointness axiom of the form DisjointnessClasses(*x*_*1*_,…, *x*_*n*_).

Notice that this propositional encoding is incomplete and, thus, the repair algorithm does not ensure that the resulting alignment is coherent. Nevertheless, in practice, the number of unsatisfiable classes present in the resulting alignment is typically small.

### ALCOMO

ALCOMO [7, 20] is an ontology alignment repair system that implements a global repair algorithm by computing the conflicting mappings before any repair selection. It has two reasoning components–an incomplete and a complete component. The first component is an incomplete pattern-based reasoning technique for detecting all minimal incoherent mapping subsets. The main idea is to first classify both input ontologies *O*_*1*_ and *O*_*2*_ using an OWL 2 reasoner. Then, given two mappings *a* ⊆ *c* and *b* ⊆ *d*, where *a* and *b*, and *c* and *d* are defined in *O*_*1*_ and *O*_*2*_ respectively, it is checked if *O*_*1*_ ⊨ *a* ⊆ *b* and *O*_*2*_ ⊨ *c* ⊆ ¬*d*. If so, it is concluded that c is unsatisfiable and thus the set of mappings is incoherent. This idea is extended and four patterns are defined that take subsumption and equivalence mappings, and properties into account. Although incomplete, this approach typically detects a large amount of conflicting mappings [11].

The second component is the application of a set of complete reasoning techniques, which are built on the classical black-box approaches for repairing ontologies (e.g. [21, 22]), over a preliminary superset of a solution, which was produced using the incomplete reasoning techniques. Thus, in this case, a coherent set of mappings is produced.

ALCOMO also takes into account the confidence values during the selection of mappings to be removed. It computes an optimal repair that removes as little confidence as possible. If all mappings have the same confidence values then an optimal repair that removes a minimum number of mappings is produced. Given the exhaustive search of this approach, a greedy approach may also be applied when dealing with large ontologies. However, since most computation effort is dedicated to the detection of conflicting mappings, ALCOMO cannot handle large ontologies efficiently.

## AgreementMakerLight Repair

Like LogMap, we use a Horn propositional logic representation of the merged hierarchy of each matched ontology with the respective alignment. Given the input ontologies *O*_*1*_ and *O*_*2*,_ the theory *T*(*O*_*1*_) (resp. *T*(*O*_*2*_)), which results from encoding the input ontology *O*_*1*_ (resp. *O*_*2*_), consists of the following Horn rules:

1. A rule *x*→*y* for all distinct classes *x*, *y* such that *x* is subsumed by *y* in *O*_*1*_ (resp. *O*_*2*_); subsumption relations are determined using an OWL 2 reasoner, and an internal reasoner.
2. A rule (*x*_*1*_∧…∧*x*_*n*_) →*y* for each subclass or equivalence axiom having the intersection of *x*_*1*_, …, *x*_*n*_ as subclass expression and *y* as superclass.
3. Rules (*x*_*i*_∧*x*_*j*_)→*false* (1 ≤ i < j ≤ n) for each disjointness axiom of the form DisjointnessClasses(*x*_*1*_,…, *x*_*n*_).
4. Rule (*x*_*i*_∧*x*_*j*_)→*false* for each pair of classes *x*_*1*_, *x*_*2*_ with incompatible cardinalities on the same data property.
5. Rule (*x*_*i*_∧*x*_*j*_)→*false* for each pair of classes *x*_*1*_, *x*_*2*_ with incompatible restrictions on the same functional data properties.
6. Rule (*x*_*i*_∧*x*_*j*_)→*false* for each pair of classes *x*_*1*_, *x*_*2*_ with incompatible restrictions on the same object properties.
  Our repair procedure is incomplete since we do not consider all the object properties relating classes. Nevertheless, as previously said, in practice, the considered object properties are usually enough to produce alignments with a small number of unsatisfiable classes [19].
  Given the ontologies *O*_*1*_ and *O*_*2*_ and a set of mappings *M* between them, we denote by *T*(*O*_*1*_∪*O*_*2*_∪*M*) the theory that results from encoding *O*_*1*_ and *O*_*2*_, as above, and *M*, by adding the following Horn rules:
7. Rule *x*→*y* for each pair *x*, *y* such that *x* ⊆ *y* is a subsumption mapping in *M*.
8. Rules *x*→*y* and *y*→*x* for each pair *x*, *y* such that *x* ≍ *y* is an equivalence mapping in *M*.

Notice that, unlike in LogMap, equivalence mappings are considered indivisible units and are not split into two subsumption mappings during the repair mapping selection.

We assume that each ontology is individually coherent and doesn't contain cycles with respect to the subsumption relation. Our repair process consists only of removing incoherence-causing mappings from the input alignment.

Our algorithms were implemented as part of AgreementMakerLight (AML) [15], a lightweight ontology matching system derived from AgreementMaker [23]. Thus, the development of our algorithms took into account the very efficient and scalable methods provided by AML to explore the relationship information of the input ontologies. For instance, the cost of checking if a class is subsumed by another class within the same ontology becomes negligible using the AML HashMap-based data structures.

## Approach

The outline of our approach to alignment repair is as follows: (1) carry out a modularization step to extract the minimal fragments from the input ontologies that allow the detection of all disjointness-based incoherences; (2) identify all mappings that cause incoherences; (3) remove a near-minimal set of mappings using an heuristic procedure.

### Ontology Modularization for Incoherence Checking

The following is our modularization algorithm, which minimizes the size of the ontology fragments that need to be analyzed within our repair setting. Notice that a set of disjoint classes denotes a set of classes {*x*_*1*_, *x*_*2*_} such that (*x*_*1*_∧*x*_*2*_)→*false*.

Definition 3: Let *O*_*1*_ and *O*_*2*_ be ontologies, *D*_*1*_ and *D*_*2*_ their respective sets of sets of disjoint classes, and *M* the alignment between them. Let *C*_*i*_ (with i∈{1,2}) be the set of classes of *O*_*i*_ that occur in *M*∪*D*_*i*_. We say that *F*_*i*_ (with i∈{1,2}) is a **core fragment** of *O*_*i*_ given *M* if *F*_*i*_ is a minimal fragment of *O*_*i*_ that satisfies the following conditions:

1. C_i_ ⊆ sigc(F_i_);
2. For every class *x* of *O*_*i*_ such that
  - there are distinct classes *y*,*z*∈*C*_*i*_ such that *x*→*y*, *x*→*z*∈*T*(*O*_*i*_); and
  - there is no class w≠*x* that for every class v∈*C*_*i*_, if *x*→*v* ∈ *T*(*O*_*i*_) then *w*→*v*∈*T*(*F*_*i*_); then *x* ∈ *sigc*(*F*_*i*_);
3. if *x*→*y* ∈ *T*(*O*_*i*_) and {*x*,*y*} ⊆ *sigc*(*F*_*i*_) then *x*→*y* ∈ *T*(*F*_*i*_).

Thus, the core fragments of a pair of ontologies given their alignment contain:

1. All classes that occur in a disjoint set, which are required for identifying incoherences, or in the alignment, which are necessary for traversing from one ontology to the other;
2. A non-redundant set of classes that contains classes that have two superclasses involved in mappings and/or disjoint sets (classes that have the same set of ancestors are considered redundant), which we call the ***checkset*** of *M*. The checkset is the set of classes that have to be checked for incoherence, as in our setting a class can only be incoherent if it has paths to two disjoint classes. Note that only one class is necessary for testing each pair of paths, and testing additional classes that have the same (or less) paths would be redundant. Moreover, since having both classes involved in a mapping in the checkset would also be redundant, the checkset contains at most one class for any given mapping. Note also that there can be different valid checksets for the same alignment repair setting, as all classes that have the same paths are valid choices for the checkset;
3. All subclass relations between core fragment classes that were modeled in the original ontologies, making it possible to traverse each ontology in search of paths to disjoint clauses.

A core fragment represents a (reduced) search space for all incoherences. The checkset denotes a (minimal) set of classes that have to be checked (search) for incoherence in the latter search space. Thus, the smaller are the core fragments and the checkset the more efficient is the incoherence detection process.

**Fig 1:**
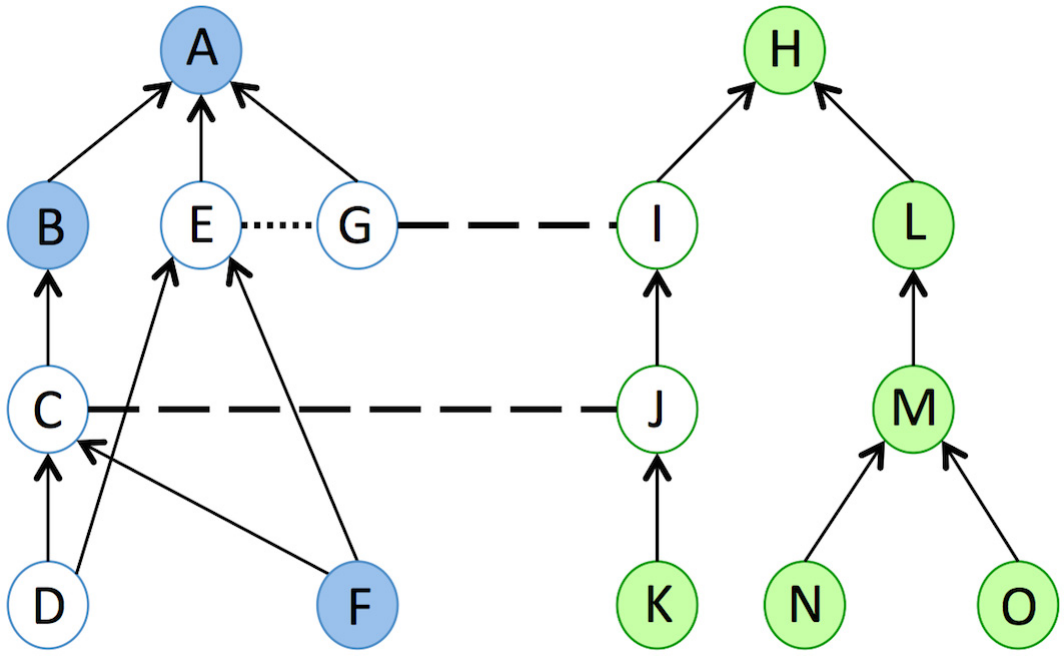
Example of an alignment between two ontologies (blue and green). Circles represent classes, arrows represent subclass relationships between classes, dashed lines represent mappings between classes, and dotted lines represent disjointness restrictions between classes. The circles filled in white represent the classes in the core fragments of the ontologies given the alignment.

Fig 1 shows an example of an alignment between two abstract ontologies, where the white circles represent computed core fragments as per Definition 1. Given Condition 1, the classes E and G were selected because they are disjoint, and the classes I, C and J were also selected because they occur in a mapping. Classes D and F satisfy Condition (2a) because they are subsumed by C and E, a mapped class and a disjoint class, respectively. However, they both have the same set of superclasses, which makes them redundant. Thus, only one of these classes will be selected (arbitrarily, class D) and it will be the checkset of the alignment, meaning it will have to be tested for coherence. Note that D is incoherent because it is a direct subclass of E and a subclass of G via the two mappings of the alignment and thus violates the disjointness restriction between E and G.

**Fig 2:**
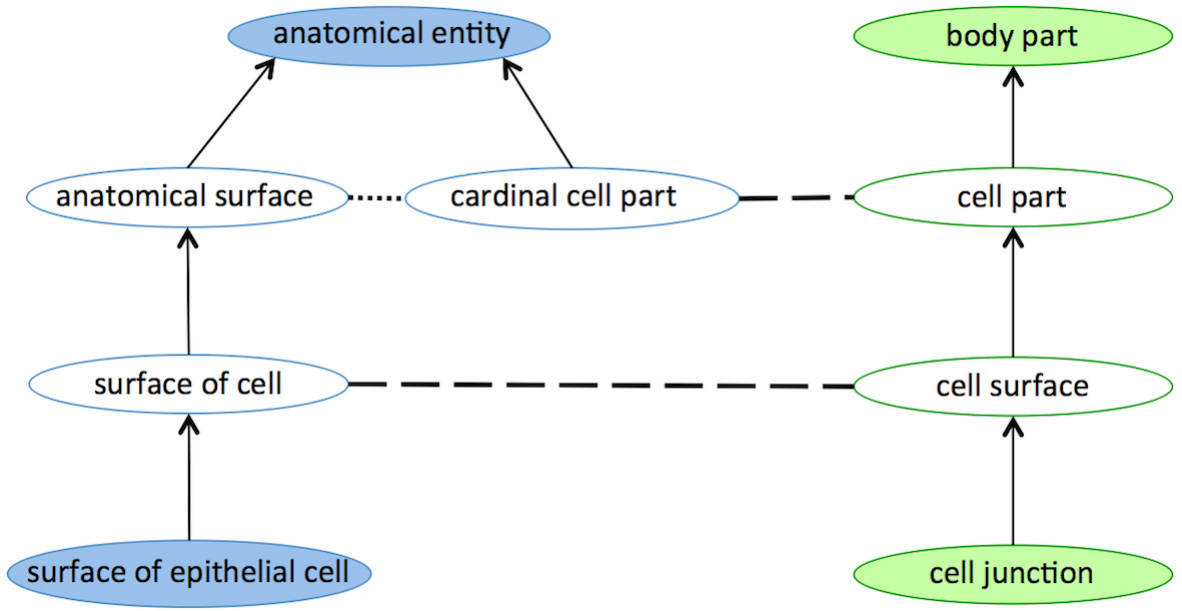
Example of two mappings taken from the UMLS-based Alignment between FMA and NCI ontologies, illustrated as blue and green respectively. Circles represent classes, arrows represent subclass relationships between classes, dashed lines represent mappings between classes, and dotted lines represent disjointness restrictions between classes. The circles filled in white represent the classes in the core fragments of the ontologies given the alignment.

Similar to Fig 1, Fig 2 shows an example of two (conflicting) mappings found in the UMLS-based alignment between FMA and NCI ontologies. A*natomical surface* and *cardinal cell part* are disjoint classes in FMA ontology. Moreover, c*ardinal cell part* and *surface of cell* FMA classes are mapped to *cell part* and *cell surface* classes from NCI ontology, respectively. Given Condition 1 of Definition 1, all the later classes belong to the core fragments of FMA and NCI.

The following proposition demonstrates that all mappings responsible for incoherences between two matched ontologies, which are captured by our Horn encoding, can be determined using core fragments.

Proposition 1: Let *O*_*1*_ and *O*_*2*_ be ontologies, *F*_*1*_ and *F*_*2*_ their respective core fragments, *M* the alignment between them, and {*x*_*1*_, *x*_*2*_} a set of disjoint classes of *O*_*1*_∪*O*_*2*_. There is a class *y* such that (*y*→*x*_*1*_)∧(*y*→*x*_*2*_) ∈*T*(*O*_*1*_∪*O*_*2*_∪*M*) if and only if there is a class *y*' such that (*y*’→*x*_*1*_) ∧ (*y*’→*x*_*2*_) ∈*T*(*F*_*1*_∪ *F*_*2*_∪*M*).

Proof: (“→”) (reductio ad absurdum) Let us assume there is a class *y* such that (*y*→*x*_*1*_) ∧ (*y*→*x*_*2*_) ∈*T*(*O*_*1*_∪*O*_*2*_∪*M*) but there is no class *y*' such that (*y*’→*x*_*1*_) ∧ (*y*’→*x*_*2*_) ∈*T*(*F*_*1*_∪ *F*_*2*_∪*M*). If (*y*→*x*_*1*_)∧(*y*→*x*_*2*_) ∈ *T*(*O*_*1*_∪*O*_*2*_∪*M*) then there must be a class *z* such that (*y*→*z*) ∈*T*(*O*_*1*_∪*O*_*2*_∪*M*) and (*z*→*p*) ∧ (*z*→*q*) ∈*T*(*O*_*i*_) where i ∈{1,2}, and *p*, *q* ∈{*x*_*1*_, *x*_*2*_} or occurs in *M*. Thus, by Condition (2) either *z* ∈ *sigc*(*F*_*1*_∪*F*_*2*_), in which case by Condition (3), (*z* →*x*_*1*_) ∧ (*z*→*x*_*2*_) ∈ *T*(*F*_*1*_∪ *F*_*2*_∪*M*), or there is a *z*' ∈ *sigc*(*F*_*1*_∪*F*_*2*_) that satisfies Condition (2a) and therefore (*z*’→*x*_*1*_)∧(*z*’→*x*_*2*_) ∈ *T*(*F*_*1*_∪*F*_*2*_∪*M*). In either case, we get a contradiction. (“←") Trivial.∎

Proposition 1 relies on the fact that, as previously stated, a class can be a subclass of two disjoint classes only if there are two distinct paths from that class that traverse the other ontology or if there is a direct path to a disjoint class and a path that traverses the other ontology and back again. In either case, there must exist a class in one of the ontologies that is a subclass of two mapped classes or a mapped class and one of the disjoint classes, which by definition will be in the checkset.

### Repair Algorithm

Our repair algorithm is described in Algorithm 1. It takes as input two ontologies, an alignment between them, and a confidence value used for filtering, and outputs a repaired alignment. Our algorithm is divided in four main tasks: (1) the computation of the core fragments (line 3); (2) the search for all conflicting sets of mappings, i.e. mappings that lead to an incoherence (line 4); (3) the resolution of incoherences using a heuristic to minimize the set of mappings removed from every conflicting set (lines 5–9); (4) an alternative resolution of incoherences by filtering the conflicting sets that have a lowest-confidence mapping and removing it from the alignment (lines 11–14).

**Algorithm 1** Description of the repair algorithm.

Procedure: Repair

**Input:** *O*_*1*_ and *O*_*2*_: ontologies; *M*: alignment to repair; *ε*: confidence threshold

**Output:** *M*’: repaired Alignment

1. *M*’: = *M*;

2. *ε*’: = *ε*;

3. (*F*_*1*_, *F*_*2*_, Checklist): = BuildCoreFragments (*O*_*1*_, *O*_*2*_, *M*’);

4. C: = ConflictSets(*F*_*1*_, *F*_*2*_, *M*’, Checklist);

5. **while** |C|>0 **do**

6. w: = SelectMappingToRemove(C, *ε*’);

7. **if** w is not null **then**

8. C: = RemoveMappings(C, {w});

9. *M*’: = *M*’ \{w};

10. **else**

11. W: = FilterConflicts(C, *ε*’);

12. C: = RemoveMappings(C, W);

13. *M*’: = *M*’ \ W;

14. *ε*’: = 1;

15. **end if**

16. **end while**

17. **return** *M*’;

#### Conflicting sets of mappings

Our implementation takes advantage of the module extraction proposed but also from the AML data structures. The ConflictSets method returns all mappings involved in an incoherence by doing a full breadth-first search in the core fragments structure for each class in the checkset. This way, we are able to determine all minimal sets of mappings, called conflicting sets, which cause the incoherences. Since conflicting sets are minimal, a conflicting set is resolved if at least one of its mappings is removed.

Formally, given ontologies *O*_*1*_ and *O*_*2*_, and a set of mappings *M* we compute the conflict sets of mappings by selecting the minimal sets from the set of all minimal sets of mappings *M*'⊆*M* such that (*y*→*x*_*1*_)∧(*y*→*x*_*2*_) ∈ *T*(*O*_*1*_∪*O*_*2*_∪*M*’) for a given checkset class *y* and disjoint classes *x*_*1*_ and *x*_*2*_.

The goal of the global repair approach is to determine a minimal set of mappings that intersect all conflict sets. This way, we are able to minimize the number of removed mappings.

#### Removing the worst mappings

Given all conflicting sets, we need to determine which mappings should be removed. The complexity of computing a global minimal set of mappings to remove, which corresponds to computing a minimal set of mappings that intersects all conflicting sets, is NP-Complete [24]. Thus, a heuristic procedure is necessary for finding a near-optimal set of mappings to remove in useful time. Our heuristic procedure consists of iteratively removing the mappings that belong to the highest number of conflicting sets (as identified by the SelectMappingToRemove method), and in case of tie, those that have the lowest confidence values. This is an efficient heuristic, which is similar to strategies applied for repairing inconsistent databases [8].

The logic behind this heuristic is that a mapping that belongs to a higher number of conflicting sets is more likely to belong to the optimal solution than a mapping that belongs to a lower number of conflicting sets. We found it to perform well empirically when the mappings removed belong to a relatively high number of conflicting sets (> 10), but less well when a lower number is reached. Thus, to complement this strategy, we propose the filtering strategy described in the following section, which is to be applied instead of this strategy when cardinality of mappings removed is below a pre-defined cardinality threshold and a confidence threshold 0 ≤ *ε* < 1 is given. In this case the SelectMappingToRemove returns null, and the remaining conflicting sets are filtered based on the value of *ε* (line 11).

### Filtering

The idea of our filtering procedure is to shift the focus of the repair process from the cardinality of the mappings to their confidence values, when the former is not sufficiently discriminative and the latter is. In conflicting sets where a mapping has a low confidence value in comparison to the other mappings in the set, we want to remove it. This means that we are more concerned with the quality of the removed mappings than with their quantity when the difference in quantity becomes small and the difference in quality is still significant. It also means that the repair strategy effectively switches from global to local because the mappings are selected to be removed within a set and not within all sets. Notice that our filtering procedure should only be applied when the input mappings have distinct confidence values.

Defining *a priori* what is a significant difference between the confidence of the mappings to apply the filtering strategy is not straightforward, given that different ontology matching systems compute confidence values differently, and with varying accuracy. Thus, we opted for using a confidence threshold *ε* as an input parameter for filtering.

Algorithm 2 describes the method FilterConflicts that computes the mappings to be filtered. It takes as input a set of conflicting sets of mappings and a confidence threshold, and it outputs a set of mappings to be removed. It is divided in two main tasks: (1) reverse ordering the set of conflicting sets by their lowest confidence mapping (line 2); and (2) selection of mappings to be removed. Task 1 ensures that we are able to minimize the number of selected mappings. If a selected mapping belongs to several conflicting sets, then all of them will be automatically resolved and there is no need to remove any more mappings from those conflicting sets (line 4). Task 2 selects the mappings to be removed by taking the mapping with the lowest confidence for each conflicting set and checking if there is no other mapping within the confidence threshold. That is, given the lowest and the second lowest confidence mappings *w*_*1*_ and *w*_*2*_, the former mapping is selected if confidence(*w*_*1*_) + *ε* < confidence(*w*_*2*_) - *ε*.

**Algorithm 2** Description of the FilterConflicts algorithm.

**Procedure:** FilterConflicts

**Input:** C: set of conflicting sets of mappings to filter; *ε*: confidence threshold

**Output:** A set of mappings to be removed.

1. W: = Ø;

2. C: = ReverseOrderByLowestConfidenceValue(C);

3. **for each** cs in C **do**

4. **if** cs ∩ W = Ø **then**

5. {*w*_*1*_, *w*_*2*_}: = LowestConfidenceMappings(cs);

6. **if** confidence(*w*_*1*_) + *ε* < confidence(*w*_*2*_) – *ε* **then**

7. W: = W∪{*w*_*1*_};

8. **end if**

9. **end if**

10. **end for**

11. **return** W;

The remaining conflicting sets, i.e. whose mappings were not selected to be removed (lines 11–13, Algorithm 1), are resolved by the method SelectMappingToRemove without any cardinality threshold (lines 14 and 6, Algorithm 1) as explained in previous section.

### Evaluation

To evaluate our approach we used the ontologies and reference alignments provided by the 2013 Ontology Alignment Evaluation Initiative (OAEI-2013) large biomedical track [25]. OAEI has been the major benchmark for ontology alignment, with the participation of state of art ontology matching systems in several ontology alignment challenges. The OAEI large biomedical track consists of finding alignments between the Foundational Model of Anatomy (FMA), SNOMED-CT (SNOMED), and the National Cancer Institute Thesaurus (NCI). These ontologies are lexically rich and contain tens of thousands of classes. The reference alignments provided are silver standard alignments based on the UMLS Metathesaurus [26] and refined using the ALCOMO debugging system in conjunction with LogMap's repair facility, and subsequent manual curation. We used OAEI-2013 reference alignments instead of OAEI-2014 to avoid any possible positive bias, since in 2014 AML was used to compute the references alignments. In our evaluation we also make use of the original unrepaired UMLS-based alignments that were used to create the silver standard alignments.

Our algorithms were implemented as part of AML and their evaluation was performed in an 8 core 16 GB server. In this section we identify the results produced by our repair implementation as AMLR. We compared our approach with the repair facility provided by LogMap 2.4, which we were able to run locally and apply in all our settings, and also with the OAEI reference alignments repaired by the latest version of ALCOMO, provided in the OAEI 2012 website.

The evaluation is divided in three parts: 1) the evaluation of our modularization approach; 2) the evaluation of our repair algorithm without filtering; and 3) the preliminary evaluation of our filtering algorithm.

To evaluate our modularization approach, we compared the sizes of the extracted modules with the sizes of the full ontologies (see Table 1) and of the modules extracted by LogMap. We also calculated the computation times of the core fragments with respect to the entire repair process.

**Table 1:** Total number of classes and disjointness axioms of the matched ontologies, the size of the of the original (unrepaired) UMLS-based alignment, and the number of incoherent classes for each OAEI large biomedical track matching problems. The number of syntactic, cardinality, functional and Object based disjointness axioms relative to the each matching task are shown in parentheses.

Matching Task	Number of Classes	Number of Disjoints	UMLS-based Alignment	Incoherent classes
FMA—NCI	145713 (78989+66724)	197 (197+0+0+0)	3024	30.590 (21.0%)^*^
FMA—SNOMED	201453 (78989+122464)	44 (44+0+0+0)	9008	78482 (39.0%)^*^
SNOMED—NCI	189188 (122464+66724)	153 (153+0+0+0)	18844	158.646 (83.9%)^**^

*Pellet reasoner was used.

**Pellet reasoner was unable to compute. ELK reasoner was used instead.

To evaluate our repair algorithm, we took the original unrepaired UMLS-based alignments, and repaired them using both AML and the repair facility of LogMap. In addition we also compared our results to the alignments repaired by ALCOMO, provided in OAEI 2012, and the alignments repaired by ALCOMO and LogMap combined, provided in OAEI 2013. We compared the alignments with regard to the number of incoherent classes, which we checked using the JENA API and Pellet OWL Reasoner [27] (except for the SNOMED-NCI task, where Pellet was unable to analyze the alignments. In this case, OWL 2 EL reasoner ELK [28] was used, which provided a lower bound on the number of unsatisfiable classes). We also compared them with regard to the total number of modifications performed over the original alignments, counting both removed mappings and altered mapping relations as modifications. This ensures that the comparison between systems that perform only mapping removal (AMLR and ALCOMO) and systems that also alter mapping relations (LogMap) is fair under the minimal impact paradigm, as both modifications have a significant cost to the alignment (*i*.*e*., potential loss of knowledge and probable misrepresentation of knowledge respectively). Nevertheless, we also discriminate the number of removed mappings in the cases where part of the modifications where altered relations.

To evaluate our filtering method, we needed alignments with confidence values, which we created by using AML both with and without background knowledge, with the same specifications as used in our OAEI 2013 entry [29]. We then repaired these alignments both using the filtering approach with a pre-defined confidence threshold of 0.05, and without using the filtering approach. We compared the performance of both repair strategies with regard to F-measure, using the OAEI 2013 repaired reference alignments.

## Results and Discussion

### Ontology Modularization for incoherence checking

Table 1 shows the number of classes and disjointness axioms for each OAEI Large Biomedical Ontologies track, the size of the respective unrepaired UMLS-based alignments that were used in this evaluation, and the number of incoherent classes found in each of these alignments. Table 2 shows the number of classes present in AMLR core fragments and checksets, and in LogMap's extracted modules, for each of the tasks in the OAEI Large Biomedical Ontologies track. Moreover, AMLR and LogMap only consider incoherencies cause by the violation of disjointness axioms (syntactic disjoints) for each of those tasks. As previously said, besides the disjointness axioms, AMLR also considers other three causes of incoherencies but they are not found in the OAEI Large Biomedical Ontologies (see Table 1).

**Table 2:** Number of classes in AMLR’s core fragments and checksets, and in LogMap's extracted modules for each matching problem in the OAEI Large Biomedical Ontologies track. The percentage of classes relative to the original ontologies is shown in parentheses.

	AML		LogMap2
	Core	Checkset	
FMA—NCI	5494 (3.8%)	2121 (1.5%)	10182 (7.0%)
FMA—SNOMED	16620 (8.3%)	6272 (3.1%)	23567 (11.7%)
SNOMED—NCI	32102 (17.0%)	16465 (8.7%)	75086 (39.7%)

It is notable that for all three matching problems, the size of the core fragments is significantly smaller than that of the original ontologies, ranging from 3.8% for the FMA-NCI task to 17.0% for the FMA-SNOMED task. Furthermore, the relatively large size of the core fragments in the latter task is mainly due to the large size of the alignment, which includes mappings between a total of 32,084 classes, and thus accounts for over 80% of the classes in the core fragments. With respect to the checksets, the results show that a much smaller percentage of the classes need to be checked for coherence, ranging from 1.5% for the FMA-NCI task to 8.7% for the SNOMED-NCI task. Thus, the core fragment modularization represents a substantial reduction of the dimension of the repair problem, both in the number of classes to check and in the search space required for checking them. In fact, without computing the core fragments and, thus, considering all classes during the search for incoherencies, AMLR was unable to complete the repair of the alignments for all matching tracks within 15 hours. This shows that computing the core fragments greatly increases the efficiency of the repair process and enables the application of a global repair strategy on large ontologies alignments.

In comparison with the extraction module implemented by LogMap, which also aims to reduce the search space, the AMLR core fragments show a considerable reduction in number of classes, particularly for the FMA-NCI and SNOMED-NCI tasks. It is worth recalling that AMLR considers more relations and properties between classes than LogMap. Thus, AMLR's core fragments represent an improvement over the extraction module implemented by LogMap. However, the relations and properties considered by AMLR but not by LogMap are not typically found in the ontologies that we currently work on. Thus, the set of disjoint classes considered by AMLR but not by LogMap is not significant (as evidenced by the fact that the alignments repaired by AMLR have the same coherency as those repaired by LogMap, see next section. Moreover, the disjoints considered are syntactic, Table 1).

Table 3 shows the core fragments computation times for each task of the OAEI Large Biomedical Ontologies track. These results show that computing core fragments represents less than 2% of the total time of the repair process (see Table 4). Furthermore, as mentioned previously, employing core fragments reduces the computation time of the repair process (by at least as much as the search space is reduced) and thus significantly increases its efficiency overall.

**Table 3:** Core fragments computation times for each OAEI large biomedical track matching problems.

Matching Task	Time (s)	% of Total Time
FMA-NCI	1	1%
FMA-SNOMED	6	1.6%
SNOMED-NCI	20	0.9%

**Table 4:** Evaluation of the repairs produced for the UMLS-based alignments from the OAEI 2013 Large Biomedical Ontologies track. Mod denotes the total number of modifications to the alignment (with removed mappings shown in parentheses when systems also alter mapping relations) and Inc denotes the number of incoherent classes.

Repair System	FMA-NCI			FMA-SNOMED			SNOMED-NCI		
	Mod	Inc	Time(s)	Mod	Inc	Time(s)	Mod	Inc	Time(s)
OAEI 2013 (ALCOMO & LogMap)	134(93)	0^*^	-	737(67)	0	-	915(368)	0^***^	-
AMLR	123	10	98	669	0	352	789	23^***^	2937
ALCOMO	205	10	^**^	876	92	^**^	-	-	-
LogMap2.4	188(140)	10	24	1058(915)	0	28	1264(914)	278^***^	151

*Manual curation was necessary to address the final 10 incoherent classes.

** Repaired alignment provided by OAEI.

***Pellet was unable to check the coherence. ELK reasoner was used instead.

To support the results obtained from each task of the OAEI Large Biomedical Ontologies track, five matching problems were selected from Bioportal [30,31] according to the size of the ontologies, number of mappings, disjoints axioms and incoherent classes; and then added to our evaluation and discussion.

The selected Bioportal matching problems are composed of alignments between Bone Dysplasia Ontology (BDO), Cell Culture Ontology (CCONT), Experimental Factor Ontology (EFO), Foundational Model of Anatomy (FMAB) (Bioportal stored version), NCI Thesaurus (NCIT) (Bioportal stored version), Online Mendelian Inheritance in Man (OMIM) and Uber Anatomy Ontology (UBERON). As shown in Tables 1–4 with respect to the OAEI Large Biomedical Ontologies track, Tables 5–8 show the stats and results with respect to the selected Bioportal problems, AMLR and LogMap repair algorithms.

**Table 5:** Total number of classes and disjointness axioms of the matched ontologies, the size of the of the original (unrepaired) Bioportal alignment, and the number of incoherent classes for each of five Bioportal matched ontologies. The number of syntactic, cardinality, functional and Object based disjointness axioms relative to the each matching task are shown in parentheses.

Matching Task	Number of Classes	Number of Disjoints	BioPortal Alignment	Incoherent classes*
BDO-NCIT	13816+105347	259 (259+0+0+0)	1636	34341 (28.8%)
OMIM-NCIT	76722+105347	196 (196+0+0+0)	5178	70172 (38.5%)
UBERON-FMAB	16854+83283	149 (117+0+32+0)	1932	4753 (4.7%)
CCONT-NCIT	17235+105347	246 (246+0+0+0)	2067	50304 (41.0%)
EFO-NCIT	14499+105347	237 (237+0+0+0)	2507	60347 (50.4%)

* ELK reasoner was used.

**Table 6:** Number of classes in AMLR’s core fragments and checksets, and in LogMap's extracted modules for five Bioportal alignments. The percentage of classes relative to the original ontologies is shown in parentheses.

	AML		LogMap2
	Core	Checkset	
BDO-NCIT	3831 (3.2%)	1742 (1.5%)	10732 (9.0%)
OMIM-NCIT	8368 (4.6%)	2449 (1.3%)	18017 (9.9%)
UBERON-FMAB	4087 (4.1%)	2156 (2.2%)	15581 (15.5%)
CCONT-NCIT	4698 (3.8%)	2049 (1.7%)	8892 (7.3%)
EFO-NCIT	5178 (4.3%)	2157 (1.8%)	23996 (20.0%)

**Table 7:** Core fragments computation times for five Bioportal Alignments.

Matching Task	Time (s)	% of Total Time
BDO-NCIT	2	1.7%
OMIM-NCIT	1	0.8%
UBERON-FMAB	2	1.7%
CCONT-NCIT	2	1.7%
EFO-NCIT	2	1.7%

**Table 8:** Evaluation of the repairs produced for the five Bioportal alignments. Mod denotes the total number of modifications to the alignment (with removed mappings shown in parentheses when systems also alter mapping relations) and Inc denotes the number of incoherent classes.

	AML			Logmap2		
	mod	Inc*	Time(s)		Inc*	Time(s)
BDO-NCIT	53	0	121	67 (59)	0	101
OMIM-NCIT	154	0	123	223 (155)	0	152
UBERON-FMAB	20	18	115	22 (17)	30	64
CCONT-NCIT	61	99	117	92 (85)	28	93
EFO-NCIT	141	3689	117	224 (191)	28	95

*ELK reasoner was used.

Notice that in Table 1, the matching problem UBERON-FMAB has functional-based disjoints axioms, which were detected by AMLR from incompatible restrictions between classes on the same functional data properties. These disjoints are not considered by LogMap.

As in Table 2 with respect to OAEI Large Biomedical Ontologies track, Table 6 shows that AMLR core fragments produce a considerable reduction in number of classes in all selected Bioportal matching problems, and, at least, 48% decrease when compared to LogMap fragments. It is worth noting that AMLR considers more 32 disjoints than LogMap in UBERON-FMAB matching problem but still produces a core fragment 48% smaller than LogMap’s.

Finally, Table 7 confirm the results obtained for the OAEI Large Biomedical Ontologies track by showing that computing core fragments represents a small percentage of the total time of the repair process (see Table 8).

### Repair

As shown in Table 4, although the degree of incoherence of the original UMLS-based alignments from the OAEI Large Biomedical Ontologies track was very high (ranging from 21.0% to 83.9%, see Table 1), all the repair systems produce alignments with a low degree of incoherence. AMLR distinguishes from the other repair systems by being much more effective in repairing those alignments, as it produces alignments with the same coherence but with less modifications.

In the FMA-NCI task, AMLR produces an alignment with 10 incoherent classes, which is thus not fully coherent. This happens because the matched ontologies include restrictions other than disjointness which are not considered by our algorithm, or by ALCOMO or LogMap. Indeed, manual curation was required to produce a coherent reference alignment for the OAEI 2013. With regard to the total number of modifications, AMLR performs only 123, whereas LogMap performs 188 (53% more), ALCOMO performs 205 (67% more), and 134 (9% more) were performed to produce the OAEI 2013 reference alignment.

In the FMA-SNOMED task, AMLR and LogMap produce a coherent alignment, while ALCOMO could not accomplish it. Once again, AMLR has the lower impact in the input alignment, by only removing 669 mappings. ALCOMO and LogMap perform 890 (33% more) and 1058 (58% more) modifications respectively, while 737 (10% more) were performed in the OAEI 2013 reference alignment.

In the SNOMED-NCI task we were unable to check the coherence of the alignments using Pellet reasoner, due to its inability to analyze these alignments. Instead we used the OWL 2 EL reasoner ELK, which provided a lower bound on the number of unsatisfiable classes. Once again, AMLR has the lower impact in the input alignment, by only removing 789 mappings, and a lower number of unsatisfiable classes, 23. LogMap perform 1264 (60% more) modifications and a higher number of unsatisfiable classes, 278. ALCOMO was unable to repair this alignment for the OAEI 2012.

In comparison with LogMap, AMLR has a significantly smaller impact on the alignments (33–60%), which is undoubtedly due to the fact that LogMap's repair algorithm is local whereas AMLR's is global. Interestingly, even if we ignore altered mapping relations and consider only the number of removed mappings, AMLR still has a smaller impact on the alignments than LogMap in all three tasks, as mapping removals account for 72–86% of LogMap's modifications. The difference between AMLR and ALCOMO (24–40% less removals) is more meaningful, given that both systems employ global repair algorithms. In comparison with the OAEI 2013 reference alignments, which combine ALCOMO and LogMap repairs, AMLR still has a smaller impact, although the difference is smaller (8–14%). In this case the number of mappings removed by AMLR is greater than those removed to produce the reference alignments, as a large fraction of the modifications done were replacements of equivalence by subsumption relations.

With respect to efficiency, AMLR took respectively 2, 6 and 49 minutes to repair the FMA-NCI, FMA-SNOMED and SNOMED-NCI alignments, including ontology loading procedures. Naturally, LogMap repairs the alignments in a much shorter time (24, 28 and 151 seconds respectively) by virtue of employing a local repair algorithm. Indeed, the comparison between the two systems highlights the key differences between local and global repair approaches, with LogMap being the more efficient system, and AMLR providing more complete alignments. Considering that alignment repair is an offline process, as no user interaction is expected, the improvement in the results is worth the time investment.

Comparing AMLR with ALCOMO would essentially reveal the gain in efficiency resulting from employing the core fragments modularization, as otherwise both systems employ global repair algorithms. Unfortunately, we were unable to repair the alignments locally in our server with ALCOMO in useful time, and opted for using the repaired alignments provided by the OAEI 2012 instead. Nevertheless, the fact that ALCOMO did not finish repairing the FMA-NCI alignment after 15 hours running in our server is already illustrative of the substantial difference in efficiency between the two systems, which again, is due to our use of the core fragments modularization.

With respect to the Bioportal matching problems, Table 8 shows that, overall, AMLR produces repairs by applying fewer modifications than LogMap. In the BDO-NCIT and OMIM-NCIT problems, both AMLR and LogMap produce coherent alignments. AMLR performs 53 and 154 modifications while LogMap performs 67 (26% more) and 223 (45% more), respectively.

In the UBERON-FMAB problem, AMLR makes 10% fewer modifications than LogMap. Since it considers more disjoints than LogMap (see Table 5), AMLR repair has fewer incoherent classes (12).

In the CCONT-NCIT and EFO-NCIT problems, AMLR performs 61 and 141 modifications while LogMap performs 92 (51% more) and 224 (59% more) modifications. Moreover, in both cases, LogMap obtains repairs with fewer incoherent classes. This is due to the fact that while modifying a much higher number of mappings to resolve detected incoherencies, LogMap also resolves undetected incoherencies accidentally.

With respect to efficiency, in four of the five BioPortal problems, AMLR took slightly more time than LogMap to repair the alignments. However, the difference is not significant considering that alignment repair is an offline process.

### Filtering

As shown in Table 9, the preliminary evaluation of our filtering method showed promising results, as the repair procedure with filtering led to an overall improvement in F-measure with only a small increase in the total number of modifications in comparison with the repair procedure without filtering. More concretely, the use of filtering led to a higher F-measure in the FMA-NCI task with both matching approaches (with and without the use of background knowledge), and in the FMA-SNOMED task with the use of background knowledge, while the F-measure of the remaining 3 alignments was the same with and without filtering. With regard to the total number of modifications, the use of filtering led to an increase ranging from 2 to 9%.

**Table 9:** Evaluation of the repairs produced with the use of filtering. F-measure and total number of modifications (Mod) of the repaired AML alignments with and without the use of background knowledge (AML-BK and AML respectively) for the tasks of the OAEI 2013 Large Biomedical Ontologies track, both with and without the use of filtering in the repair procedure.

Matching Task	Alignment	No Filtering		Filtering	
		F-measure	Mod	F-measure	Mod
FMA-NCI	AML	80.2%	64	**80.5%**	66
FMA-NCI	AML-BK	80.3%	74	**80.4%**	79
FMA-SNOMED	AML	75.7%	294	75.7%	300
FMA-SNOMED	AML-BK	76.2%	325	**76.3%**	336
SNOMED-NCI	AML	71.1%	298	71.1%	324
SNOMED-NCI	AML-BK	71.8%	300	71.8%	328

It is important to note that we are evaluating the F-measure of our approach with respect to the OAEI 2013 reference alignments, which were repaired automatically using ALCOMO and LogMap. Thus, the improved F-measure using filtering may be due to the fact that this repair algorithm produces results that are more similar to the combination of ALCOMO and LogMap than the repair algorithm without filtering [6]. It does not necessarily mean that the alignments produced with filtering are more correct, as the combination of ALCOMO and LogMap could potentially lead to less correct alignments than our repair algorithm without filtering. However, ascertaining this would only be possible through manual evaluation. Thus, further evaluation will be necessary to fully assess the impact of our filtering strategy. Nevertheless, given that the filtering is only applied when the main selection heuristic is expected to be less accurate, and that its effect on the total number of modifications is small, we don't expect its impact to lead to substantially worse results than those obtained without filtering, even when the confidence values are unreliable.

We know *a priori* that the accuracy of our filtering method is closely related to the reliability of the confidence values provided by the ontology matching system. If these values are not well correlated to the correctness of the mappings, the filtering process will be inaccurate. For instance, in the case of AML (which uses mainly lexical matching strategies) the presence of homonyms that are not true synonyms in the matching problem will compromise the reliability of the confidence values. Cases such as this highlight the importance of profiling the input ontologies to optimize the matching and/or repair strategies [32].

Finally, with regards to efficiency, the filtering method adds a marginal complexity cost to the main repair algorithm due to the set sorting step. However, this is compensated by the switch from global to local mapping selection, which leads to a small reduction in the overall computing time.

### Participation in OAEI

AML participated in the last two editions of OAEI. In OAEI 2013, AML participated with the first version of AMLR and obtained top places in the anatomy and large biomedical tracks. AML and LogMap efficiently produced alignments with the lowest degree of incoherence in all main tracks (less than 0.1%). However, in some tracks, AMLR computed alignments with a lower number of mappings and obtained a lower value of F-measure than LogMap. This was due to the fact that LogMap splits equivalence mappings into two subsumption mappings and it was used, together with ALCOMO, to repair the reference alignments. Based on the conflict sets computed by AMLR, [6] we identified the bias in these reference alignments and proposed a new evaluation method which ignores the mappings that belong to a conflict set. This issue was addressed by OAEI and the proposed evaluation was implemented in the Large BioMed Track in its latest edition (2014) by using AMLR in the creation of a new reference alignment.

In OAEI 2014, AML participated with the latest version of AMLR obtaining the first place and the lowest degree of incoherence (average of 0.0045%) in the large biomedical ontologies track.

## Conclusions and Future Work

We have shown that our repair algorithm is effective, as it produced alignments with the same degree of incoherence as state-of-the-art repair systems, ALCOMO and LogMap. This demonstrates that, at least for the ontology matching problems evaluated, the simplification of looking only at disjoint and some property restrictions is a suitable approximation. While we believe that it will also be a suitable approximation for most practical repair problems, it is possible to extend our algorithm to account for additional restrictions, which we will pursue in future work.

We have also shown that our algorithm performs better than other repair systems under the paradigm of minimal impact, as it made fewer modifications to the alignments than ALCOMO or LogMap. The difference between our algorithm and LogMap's was expected considering that the former is global and the latter is local. However, it is worth noting that even if we ignore LogMap's altered mapping relations and count only removed mappings, our algorithm still made less modifications than LogMap. Thus, considering the impact of altered mapping relations [6], our algorithm produced alignments that were both more complete and semantically more correct than those produced by LogMap. Regarding the difference between our algorithm and ALCOMO's, given that both are global, it validates our selection heuristic and suggests that it approximates the global optimum more closely than ALCOMO's.

With regard to efficiency, we have shown that our algorithm is significantly more efficient than ALCOMO, taking less than 2 minutes to perform a task that ALCOMO was unable to complete in 15 hours. This difference in efficiency is tied to the main innovation of our repair algorithm, which is the core fragments modularization that reduces the size of the repair problem and mitigates the scalability issue.

Our evaluation showed that this modularization was highly effective, reducing the repair problem to at most 17% of its original size, and improving significantly upon the modularization implemented by LogMap. Indeed, without the core fragments modularization, the quality of the results of our repair algorithm would not be possible in useful time. In spite of our more effective modularization, our repair algorithm is less efficient than LogMap overall, given that being local, the repair algorithm of the latter is computationally much less demanding than ours. Nevertheless, we believe that the improved quality of our repair algorithm with regard to the impact on the alignments is worth the additional time investment.

The top places obtained in OAEI 2013, our important contribution and outstanding results in OAEI 2014 strengthen the validity of our approach and its results. Moreover, AMLR also took part in a case study of BioPortal [19], in which it produced repairs with a low degree of incoherency and impact for all the 11 (incoherent) alignments studied. It also contributed to identify a high percentage of previously unknown incoherencies among the mappings stored in BioPortal. A solution was proposed to annotate BioPortal mappings with information about their logical conflicts. The evaluation made for the OAEI Large Biomedical Ontologies track was expanded with five matching problems from the Bioportal case study. The new results confirmed the outcomes obtained for the OAEI Large Biomedical Ontologies track.

In addition to our main repair algorithm, we presented a variant thereof, which applies a filtering method as an alternative to the selection heuristic when the discriminating power of the latter becomes low. The filtering method shifts the focus of the repair from minimizing the number of removed mappings to minimizing the confidence of each removed mapping, so it corresponds to a local repair strategy. This means that this variant of our repair algorithm is hybrid, combining global and local repair. While the preliminary evaluation of this repair variant was inconclusive, due to the limitations of the reference alignments available, we believe that this combination of strategies is promising and deserves a more in-depth evaluation. Indeed, in future work, we will be exploring this combination strategy.

AML (AMLR) is open-source and is freely available in GitHub (https://github.com/AgreementMakerLight) both as a standalone GUI tool and as an Eclipse project.
